# Cell Cycle Changes after Glioblastoma Stem Cell Irradiation: The Major Role of RAD51

**DOI:** 10.3390/ijms19103018

**Published:** 2018-10-03

**Authors:** Gaelle Tachon, Ulrich Cortes, Pierre-Olivier Guichet, Pierre Rivet, Anais Balbous, Konstantin Masliantsev, Antoine Berger, Odile Boissonnade, Michel Wager, Lucie Karayan-Tapon

**Affiliations:** 1 Laboratoire de Neurosciences Expérimentales et Cliniques (LNEC), Institut National de la Santé et de la Recherche Médicale (INSERM) Unité 1084, Université de Poitiers, F-86073 Poitiers, France; pierre-olivier.guichet@chu-poitiers.fr (P.-O.G.); anais.balbous-gautier@chu-poitiers.fr (A.B.); k.masliantsev@chu-poitiers.fr (K.M.); m.wager@chu-poitiers.fr (M.W.); l.karayan-tapon@chu-poitiers.fr (L.K.-T.); 2 Département de Cancérologie Biologique, Centre Hospitalo-Universitaire de Poitiers, F-86021 Poitiers, France; u.cortes@chu-poitiers.fr (U.C.); labonco.chupoitiers@gmail.com (P.R.); 3 Faculté de Médecine-Pharmacie, Université de Poitiers, F-86021 Poitiers, France; 4 Département d’Oncologie Radiothérapie, Centre Hospitalo-Universitaire de Poitiers, F-86021 Poitiers, France; a.berger@chu-poitiers.fr (A.B.); odile.boudinot@chu-poitiers.fr (O.B.); 5 Département de Neurochirurgie, Centre Hospitalo-Universitaire de Poitiers, F-86021 Poitiers, France

**Keywords:** cell cycle, DNA repair protein RAD51 homolog 1 (RAD51), Glioma Stem Cells, ionizing radiation

## Abstract

“Glioma Stem Cells” (GSCs) are known to play a role in glioblastoma (GBM) recurrence. Homologous recombination (HR) defects and cell cycle checkpoint abnormalities can contribute concurrently to the radioresistance of GSCs. DNA repair protein RAD51 homolog 1 (RAD51) is a crucial protein for HR and its inhibition has been shown to sensitize GSCs to irradiation. The aim of this study was to examine the consequences of ionizing radiation (IR) for cell cycle progression in GSCs. In addition, we intended to assess the potential effect of RAD51 inhibition on cell cycle progression. Five radiosensitive GSC lines and five GSC lines that were previously characterized as radioresistant were exposed to 4Gy IR, and cell cycle analysis was done by fluorescence-activated cell sorting (FACS) at 24, 48, 72, and 96 h with or without RAD51 inhibitor. Following 4Gy IR, all GSC lines presented a significant increase in G2 phase at 24 h, which was maintained over 72 h. In the presence of RAD51 inhibitor, radioresistant GSCs showed delayed G2 arrest post-irradiation for up to 48 h. This study demonstrates that all GSCs can promote G2 arrest in response to radiation-induced DNA damage. However, following RAD51 inhibition, the cell cycle checkpoint response differed. This study contributes to the characterization of the radioresistance mechanisms of GSCs, thereby supporting the rationale of targeting RAD51-dependent repair pathways in view of radiosensitizing GSCs.

## 1. Introduction

Glioblastoma (GBM) is the most malignant type of primary brain tumor [1]. After initial diagnosis of GBM, standard treatment consists of maximal surgical resection, radiotherapy, and concomitant adjuvant chemotherapy with temozolomide (TMZ) [2]. However, the median time to recurrence is 6.9 months, and the median survival time from diagnosis is 14.6 months [3]. Many GBMs initially respond to treatment, but residual radioresistant cells often survive after radiotherapy and give rise to tumor recurrences. Attempts to improve patient outcomes by increasing radiation doses have been unsuccessful due to necrosis in the surrounding brain, and diffuse cerebral atrophy, as a consequence of exposing healthy cells to levels exceeding their dose tolerance limit [4]. A more efficient strategy would be to suppress or overcome the mechanisms underlying radioresistance in conjunction with radiotherapy.

Several studies have highlighted the presence in the tumor of a subpopulation of cells with characteristics similar to the neural progenitor cells called “Glioma Stem Cells” (GSCs) [5,6]. A pool of these cells can survive exogenous DNA damage, such as radiation-induced double-strand breaks, and repopulate the tumor following treatment, thereby contributing to radioresistance and tumor recurrence [7]. It is now acknowledged that GSCs are highly resistant to chemotherapeutic drugs, including TMZ, and promote radioresistance by preferential activation of a DNA damage response [8]. Initial characterization of the radioresistant properties of GSCs has shown that these cells may resist radiotherapy through preferential activation of a DNA damage checkpoint response and increased DNA repair capacity [8]. Other studies have reported cell cycle defects in the G1/S and S phase checkpoints following irradiation, suggesting that homologous recombination (HR) and cell cycle checkpoint abnormalities may contribute concurrently to the radioresistance of GSCs [9,10]. Existing models established in different cell types have shown that radio-induced double-strand breaks (DSB) undergo faster repair in the G2 phase during the first hours following irradiation [11]. Ropolo et al. indicated that GSC lines display a significantly elongated cell cycle compared to non-stem cells in response to enhanced activities of the DNA damage checkpoint kinases Chk1 and Chk2 [12]. Several other studies have reported that the sensitivity of cells to IR varied widely depending on the phase of the cell cycle at the time of irradiation, and have also shown that cells in the G2 and M phases were approximately 3 times more sensitive than cells in the S phase [13,14,15].

Overexpression of RAD51 in cancer has been widely documented, particularly in glioblastoma [16]. RAD51 protein is crucial for HR, and its inhibition has been shown to sensitize GSCs to irradiation or alkylating drugs [17,18]. RAD51 expression is closely related to the state of cell proliferation and maximally transcribed in the late S and G2 phases [19,20]. In a previous paper, we analyzed the DNA damage response after ionizing radiation (IR) in a panel of 10 GSC lines derived from patients with clearly defined molecular characteristics [21]. While our results underscored efficient DNA repair in all GSC lines, due to variations of intrinsic radioresistance the responses were heterogeneous. Indeed, GSCs could be divided into two groups according to their DNA repair kinetics at 4Gy: a radiosensitive group including GSC-1, -3, -5, -10, and -11, and a radioresistant group including GSC-2, -6, -9, -13, and -14. This study highlights the importance of RAD51 expression in GSCs and, more specifically, of the HR pathway involved in radioresistance. It should consequently be of interest to characterize cell cycle progression and cell cycle changes following RAD51 inhibition in GSCs. The aim of this study was to examine the consequences of IR for cell cycle progression in GSCs. In addition, we intended to assess the potential effect of RAD51 inhibition on cell cycle progression in GSCs.

## 2. Results

### 2.1. Analysis of Radioresponse and Cell Cycle Progression of GSCs

**Figure 1:**
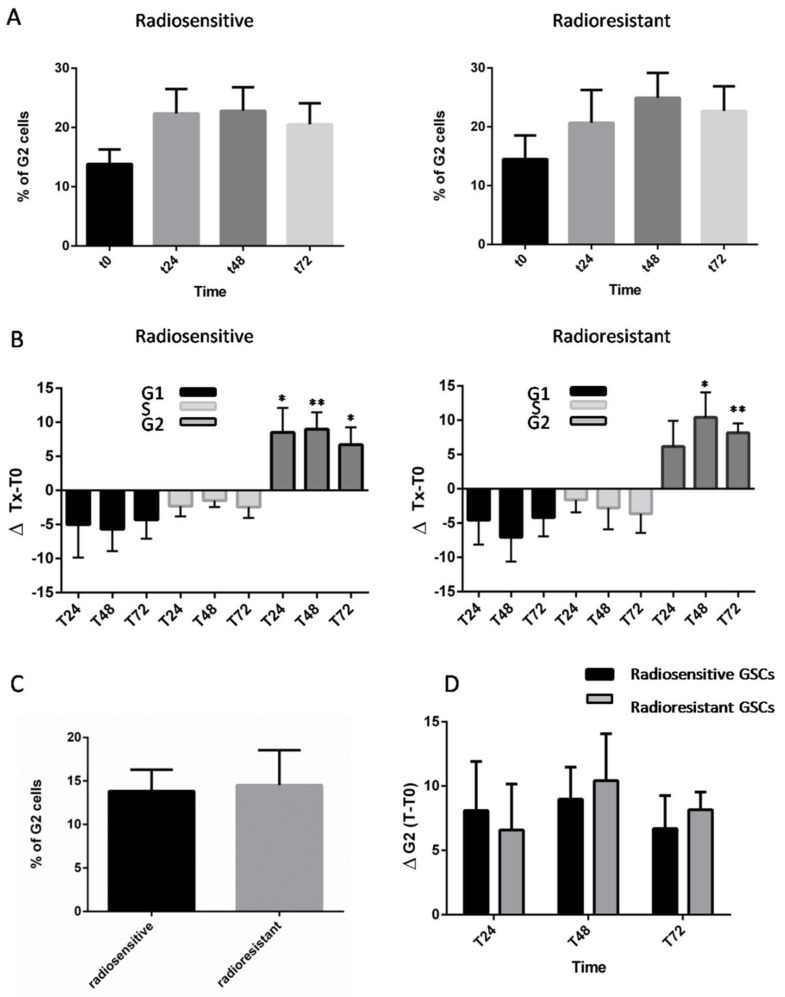
Analysis of cell cycle progression of radiosensitive and radioresistant unsynchronized Glioma Stem Cell (GSC) lines after 4Gy IR exposure (merged data are shown). (**A**) Proportion of cells in G2 phase after 0, 24, 48, and 72 h following IR. Histograms represent mean data ± the standard error of the mean (SEM, Mann–Whitney test). (**B**) Mean variations of G1, S, and G2 phases following IR. (* *p* < 0.05; ** *p*  < 0.01; Kruskal–Wallis test). (**C**) Comparative proportion of cells in G2 phase at T0 before IR between radioresistant and radiosensitive cell lines; Histograms represent the mean data ± SEM. (**D**) Mean variation of G2 phase at 24, 48, and 72 h post-irradiation in radiosensitive and radioresistant GSCs.

Ten GSC lines were subjected to 4Gy IR and we evaluated cell cycle progression through fluorescence-activated cell sorting (FACS) analysis. Variations in cell cycle phases were compared at 24, 48, and 72 h. A significant increase of cell populations in the G2 phase was observed among all GSCs concomitantly with a decrease of G1 and S phase fractions (Figure 1A,B, Figures S1 and S2). Analysis of later time points indicates that all GSCs were able to sustain G2 arrest for 72 h. In this study, we compared G2 populations between the two groups in the absence of IR (T0) and found no statistical difference (Figure 1C). We then compared mean variations in G2 phase following IR (ΔG2), and once again no significant difference was observed between the two groups (Figure 1D).

### 2.2. Synchronization and Cell Cycle Progression of GSCs

**Figure 2:**
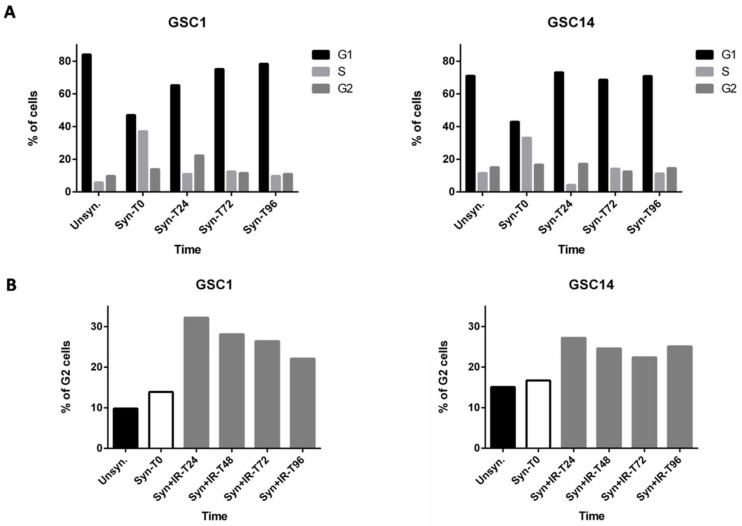
Analysis of cell cycle progression in GSC-1 and GSC-14 lines and the effect of IR. (**A**) Mean variation of cell cycle phases in GSC-1 and GSC-14 after synchronization. GSCs were synchronized by a double thymidine block, released, and collected at the indicated time points (Unsyn: unsynchronized, Syn: synchronized). (**B**) Proportion of synchronized cells in G2 phase following 4Gy IR. Cells were collected at the indicated time points. (Unsyn: unsynchronized without IR, Syn: synchronized without IR, Syn+IR: synchronized with IR). Histograms represent the mean data ± SEM obtained for both cell lines.

In unsynchronized cultured GSCs, populations at different stages of the cell cycle can co-occur. In this respect, FACS analysis has demonstrated that, in untreated GSCs, the G2 phase population ranged from 4.8 to 27.2% (Figure S2), and that these differences may markedly modify their radiosensitivity. To circumvent this problem and to confirm that the previously observed G2 arrest was not biased, we synchronized GSC cultures before IR exposure. Following a double thymidine block, GSC-1 and GSC-14 were efficiently synchronized at the G1/S border with an almost equal distribution of cells in the G1 and S phases (Figure 2A). After removal of thymidine (T0), cells re-entered into the cycle over the next 24 h, thereby underscoring the reversibility of the G1/S block (Figure 2A). As was previously observed with unsynchronized cells, IR induced an increase in the G2 phase population of synchronized GSCs (Figure 2B).

### 2.3. Analysis of Radioresponse of GCSs according to the Verhaak Classification

**Figure 3:**
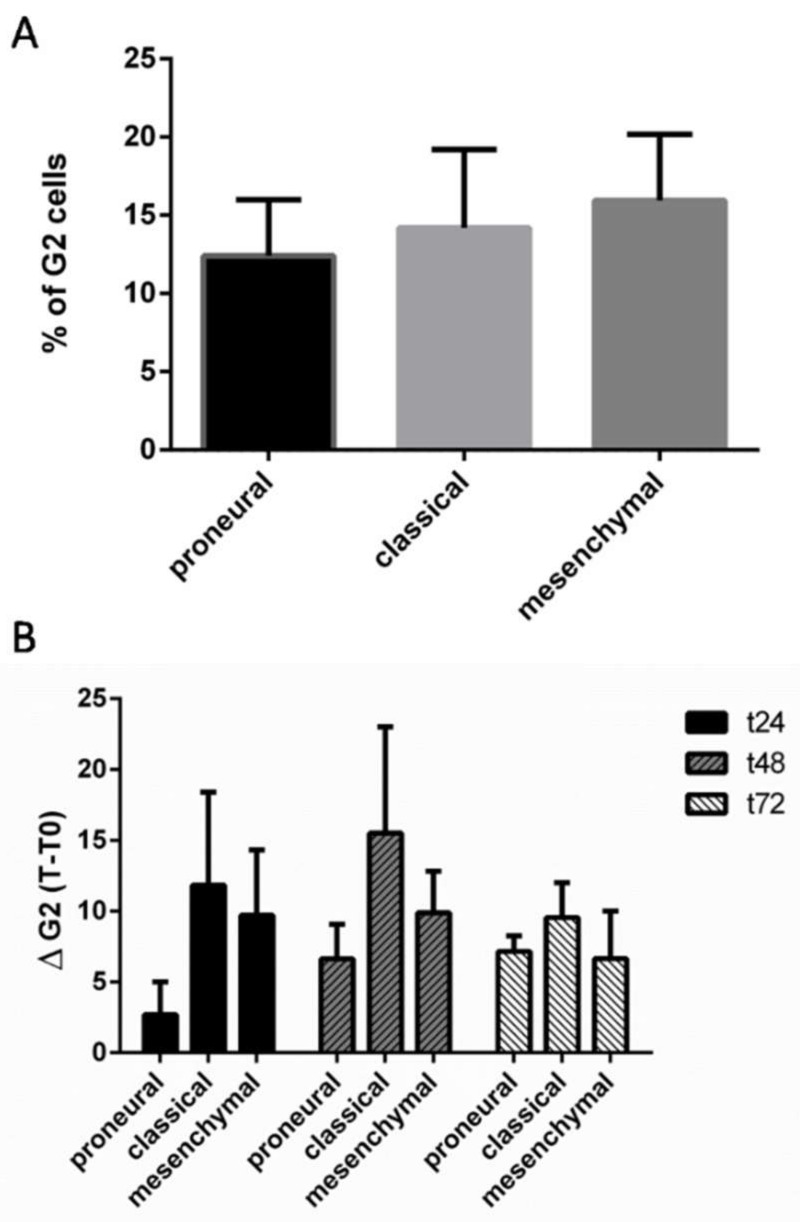
G2 phase variation in GSC lines according to the Verhaak classification scheme (merged data are shown). (**A**) Comparative proportion of cells in G2 phase at T0 before IR. (**B**) Mean variation of G2 phase in GSCs following 4Gy IR. Cells were collected at the indicated time points. Histograms represent the mean data ± SEM. Proneural (*n* = 4), Classical (*n* = 2), and Mesenchymal (*n* = 4).

We first classified GSCs according to the Verhaak signature using an Agilent SurePrint G3 Human GE (8 × 60K) chip. Centroid-based correlation analysis indicated that GSCs could be divided into three subtypes (proneural, classical, and mesenchymal) (Table S1). Both radiosensitive and radioresistant groups of GSCs presented an equal distribution of proneural, classical, and mesenchymal Verhaak subtypes (Figure S2). We then evaluated G2 populations of GSC lines before IR and found no significant differences in G2 phase distribution between the subgroups (Figure 3A). Following IR, proneural subtype presented reduced G2 arrest compared to other Verhaak subtypes, but the difference was not statistically significant (Figure 3B).

### 2.4. The Consequences of RAD51 Inhibition for GSC Radioresponse and Cell Cycle Progression

**Figure 4:**
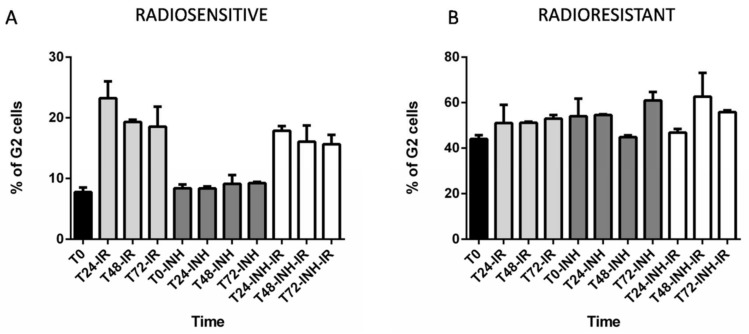
Effect of RI-1 (10 µM) inhibitor on cell cycle progression of the GSC-1, GSC-6, GSC-11, and GSC-14 lines with or without IR exposure (merged data are shown). (**A**,**B**) Mean variation of G2 phase of radiosensitive GSC-1 and GSC-11 cells (**A**) and radioresistant GSC-6 and GSC-14 cells (**B**) after 4Gy exposure with or without RI-1. Cells were collected at the indicated time points (IR: irradiation, INH: RI-1 inhibitor). Histograms represent the mean data ± SEM.

Targeting RAD51-dependent repair is known to enhance tumor cell radiosensitivity [21,22,23,24]. In the present study, we assessed the effect of RAD51 inhibition on cell cycle profiles by using RI-1 inhibitor in radioresistant (GSC-6 and GSC-14) and radiosensitive cell lines (GSC-1 and GSC-11), with or without IR exposure. In radiosensitive cell lines, RI-1 did not modify G2 phase progression whereas radioresistant GSCs exhibited a more substantial increase of G2 population, aside from at the time point 48 h (Figure 4A,B). FACS analysis of radiosensitive GSC lines (GSC-1 and -11) showed major G2 arrest after 4Gy IR that was moderately inhibited by RI-1 inhibitor (Figure 4A). A differential effect of RI-1 inhibitor was noted on radioresistant cell lines (GSC-6 and -14) as it increased the G1 fraction at 24 h post-irradiation (T24-INH = 40% versus T24-INH-IR = 49%) and delayed G2 arrest post-irradiation by up to 48 h (Figure 4B). Finally, we subjected radioresistant GSC-14 cells to a major radiation dose of 16Gy and, similarly to radiosensitive cell lines, GSC-14 presented a non-delayed G2 checkpoint after a high dose of IR, independently of RI-1 inhibitor (Figure S3).

### 2.5. GSC Differences and Gene Expression

In the previous [21] and current study, we showed that the two groups differed according to their RAD51 protein expression, their repair kinetics, and their cell cycle profiles post-IR +/− RAD51 inhibition (Table 1).

**Figure 5:**
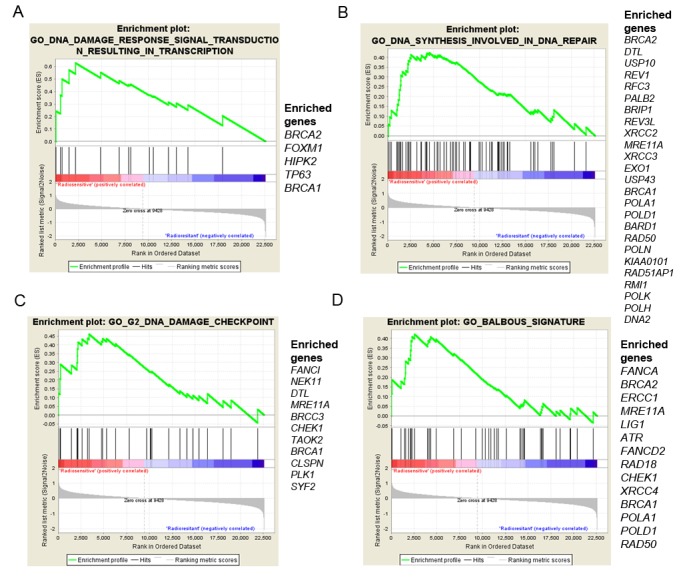
Comparison of gene expression enriched in radiosensitive GSCs to radioresistant GSCs. (**A**) Significant enrichment of a signature of 15 genes involved in DNA damage response (*p* value = 0.003, False Discovery Rate (FDR) = 0.072. Normalized Enrichment Score (NES) = 1.849). (**B**) Significant enrichment of a signature of 71 genes involved in DNA repair (*p* value < 0.001, FDR = 0.0369, NES = 1.847). (**C**) Signature of 32 genes involved in the DNA damage checkpoint (*p* value = 0.01, FDR = 0.0839, NES = 1.691). (**D**) Signature of 46 genes involved in DNA repair (*p* value = 0.013, FDR = 0.0838, NES = 1.661).

We then performed microarray analysis to investigate the differential expression of genes between the two groups in order to better understand the differences observed in cell cycle responses to IR. We performed gene set enrichment analysis between GSCs 1–3–5–10–11 and GSCs 2–9–6–13–14 according to the C5.bp.v6.1 gene set comprising 4436 gene ontology (GO) terms and according to a target gene set involved in DNA damage checkpoints, the cell cycle, or DNA repair. We found significant enrichment of DNA damage checkpoint and DNA repair process signatures (Table S2). Only the four most illustrative signatures with a Normalized Enrichment Score (NES) ≥1.66 and an False Discovery Rate (FDR) < 0.1 with the list of enriched genes are shown in Figure 5. Interestingly, genes from the Fanconi Anemia pathway *FANCA*, *FANCI*, *FANCD1* (also known as *BRCA2*) were enriched in radiosensitive GSCs compared to radioresistant GSCs (Table S2).

We then performed a Taqman Low density array (TLDA) assay to compare the expression of 46 target genes involved in DNA damage checkpoints, the cell cycle, or DNA repair between the radiosensitive and the radioresistant groups. Without IR exposure, significantly higher *FANCD2* expression was observed in the radiosensitive group (BALBOUS signature, Table S2).

## 3. Discussion

HR and cell cycle checkpoint abnormalities can contribute to the radioresistance of GSCs and the targeting of both may represent a potential alternative treatment therapy for GBM patients. However, controversial issues have arisen about resistance phenotypes of GSCs to DNA-damaging agents and IR [8,25,26]. As a consequence of genetic heterogeneity in cancer, GSCs isolated from different patients may differ in their responses to DNA damage and, particularly, in cell cycle checkpoint occurrence [27]. To our knowledge, this study is the first attempt to characterize cell cycle progression and cell cycle changes following RAD51 inhibition in GSCs and to evaluate its potential as a new therapeutic target.

We first assessed cell cycle changes in 10 GSC lines derived from patients with clearly defined molecular characteristics [21,28,29]. Following 4Gy IR, all GSCs displayed a significant increase of cell populations in G2 phase at 48 h, which was persistent for up to 72 h. This finding was consistent with results from Wang et al. showing prominent S- and G2-phase checkpoints in response to IR in embryonic carcinomas [30]. In glioma-initiating cells, Lim et al. described a defect in cell-cycle arrest at the G_1_/S-phase and low dependency on the Non-Homologous End Joining (NHEJ) repair pathway following DNA damage [10].

DSB repair pathways have overlapping roles in DNA repair and are highly dependent on cell cycle phase [31]. Differential cellular sensitivities to IR have been reported depending on the phase of the cell cycle at the time of irradiation [13]. Hence, cells are known to be more radioresistant in the early G1 and late S phases and show increased radio sensitivity in the early S phase [14,15]. Synchronization of GSCs confirmed that a G2 checkpoint is preferentially used in these cells in response to radiation-induced DNA damage and that this outcome is independent of initial cell cycle phase.

It has been acknowledged that HR is active only in the postreplicative stages of the cell cycle, S and G2, during which time the sister chromatide is available [32]. In addition, rather than NHEJ, HR is preferentially activated in GSCs throughout the S/G2 checkpoint in response to DNA damage [33,34]. The G2 arrest observed in all GSC lines in response to IR may then be concomitant with HR activation.

RAD51 is a major component of HR-mediated DNA repair and is overexpressed in many cancers [35,36,37], including glioblastoma [16]. RAD51 activity is partially cell-cycle-dependent, with higher expression in the G2 phase in various cell types [20]. We previously identified two groups of GSCs based on their comet assay profiles with a radioresistant group that showed a significant increase in RAD51 protein expression after IR [21] (Table 1). In this current study, all GSC lines tested displayed a G2 checkpoint response after 4Gy IR, thereby underscoring the importance of G2 arrest in GSC response to DNA damage and supporting the major role of RAD51 in the repair mechanism, at least in the radioresistant group.

The radioresistant set of cell lines also presented a significant decrease in DNA repair after RAD51 inhibition, leading to increased cell death [21] (Table 1). Several attempts to target RAD51-dependent repair have increased the sensitivity of tumor cells to radiotherapy, albeit to a variable extent [22,23,24]. A recent publication confirmed the importance of RAD51 in radioresistance mechanisms, highlighting the potential of RAD51 as a means of targeting specifically radiosensitized GSCs [18]. However, so far, there have been no published reports evaluating the cell cycle profile in GSCs and RAD51 inhibition.

After 4Gy IR, the radioresistant group displayed different G2 profiles compared to radiosensitive cells. Addition of RI-1 inhibitor had only a small influence on the cell cycle of radiosensitive cells, with a slight decrease in G2 populations.

This observation may reflect other repair pathways acting in the radiosensitive group requiring G2 arrest and, to a lesser extent, RAD51. In contrast, radioresistant cells manifested an increased G1 fraction and delayed G2 arrest by up to 48 h in the presence of RI-1. Recently, Chen et al. highlighted a significant time lapse in the cell cycle transition at the G1/S phase using the same RI-1 inhibitor in cervical cancer cells [38]. Thus, the G2 delay observed in radioresistant GSCs after IR and RI-1 treatment might be caused, at least to some extent, by attenuation of the cell cycle transition from G0/G1 to S phase. One of the limitations of this study was the slow doubling time of GSCs (mean doubling time: 7 days), and as the experiment was consequently focused on four independent cell lines representative of the radioresistant and radiosensitive groups, statistical analysis was not possible.

In the presence of RI-1, it is worth noting that radioresistant cells exhibit higher apoptosis and necrotic indexes that, in addition to our cell cycle results, are consistent with the central role of RAD51 in DNA damage response and radioresistance, thereby corroborating previous studies [39,40,41] (Table 1).

This study highlighted significant variation in G2 populations at T0 (Figure S2 versus Figure 4). In this regard, plasticity of glioma stem cells is a well-known phenomenon that has evolved overtime as a consequence of genetic heterogeneity, generating subclones with differential responses to environmental changes [42]. As basal levels of G2 populations can vary widely in unsynchronized glioma stem cells, variations were taken into consideration when designing our experiments to monitor the cell cycle changes under exposure to IR and other chemicals. In this study, changes in cell cycle population were evaluated after adjusting to the T0 time point. Furthermore, additional synchronization experiments showed that G2 arrest was independent of the initial cell cycle state.

It is recognized that GSCs isolated from different cell lines or patients might present genetic heterogeneity, which could explain differences in the radiosensitive thresholds triggering DNA damage response and repair, as well as differences in cell cycle profiles [27,43]. We studied differences between molecular subtypes according to the Verhaak classification, one of the most widely acknowledged molecular glioblastoma classifications, and nevertheless found the reduced G2 arrest after IR in the proneural subtype not to be significant. GSCs differed according to their intrinsic radioresistance, with higher expression of RAD51 in radioresistant cells after IR exposure. Gene Set Enrichment Analysis (GSEA) revealed significant enrichment of Fanconi anemia pathway genes in the radiosensitive group in comparison with the radioresistant group, and, interestingly, no *RAD51* enrichment was pointed out. A TLDAassay was used to compare the expression of 46 target genes involved in DNA repair and confirmed *FANCD2* enrichment in the radiosensitive group. The *FANCI–FANCD2* complex is known to stabilize RAD51–DNA interaction [44], and direct binding of *FANCD1* to *RAD51* through the *BRC* repeat domain is well-established [45]. Therefore, this observation could be indicative of impaired activity of Fanconi anemia genes in radioresistant as opposed to radiosensitive GSCs.

Indeed, Schlacher et al. reported that overexpression of *RAD51* in *FANCD2*-deficient cells can partially rescue replication fork instability and secure fork protection [46,47]. These results are consistent with the increased RAD51 expression observed in radioresistant GSCs after IR [21] and support the assumption that radioresistant cells rely on RAD51 for DNA damage response, whereas radiosensitive GSCs may tend to favor other repair mechanisms. Cell cycle checkpoints and mechanisms involved in DNA repair are clearly interdependent, with the choice of repair mechanism consistently adjusted throughout the cell cycle [34]. Herein, the significant difference observed in repair gene expression between the two groups may to a certain extent explain the differences observed in cell cycle profiles.

To conclude, these data should enhance our understanding of the mechanisms governing radioresistance in GSCs and support the rationale for targeting RAD51 repair pathways in view of radiosensitizing GSCs.

## 4. Methods

### 4.1. GSC Lines and Cell Culture

Glioblastoma stem cell cultures were derived from freshly resected tumors after written informed consent obtained from each patient enrolled in the study. This study was approved the 30/06/2004 by the ethics committee of Poitiers University Hospital (DHOS/OPRC/FCnotif-tumoro-jun04: 04056), in accordance with the Declaration of Helsinki. The cells were grown as previously described in [18,19,20]. Briefly, GSCs were cultured at 37 °C as proliferative non-adherent spheres in Neurobasal medium (NBE, Life Technologies, Carlsbad, CA, USA) supplemented with 20 ng/mL of basic fibroblast growth factor (bFGF, Life Technologies), 20 ng/mL of epidermal growth factor (EGF, Life Technologies), and culture supplements N2 (100×, Life Technologies) and B27 (50×, Life Technologies). Culture medium was replaced twice a week and, when the spheres became large, they were enzymatically dissociated with accutase (Merck-Millipore, Billerica, MO, USA). GSCs have previously been characterized as regards self-renewal, differentiation, and in vitroclonogenicity by limiting dilution assays [28,29]. Tumorigenicity was evaluated by xenograft experiments in nude mice. The molecular traits of the GSCs were detailed in Balbous et al. [21].

### 4.2. Cell Irradiation

Gamma irradiation was performed at the Department of Radiotherapy (University Hospital of Poitiers, Poitiers, France) with an Elekta Synergy Beam Modulator (dose rate, 4.56Gy/min). GSCs were irradiated at room temperature in tissue culture flasks and cultured at 37 °C. Control cells were subjected to the same experimental conditions.

### 4.3. G1/S Cell Synchronisation with a Double Thymidine Block

As regards GSC synchronization at the G1/S border, the double thymidine procedure was chosen due to the fact that thymidine is an easily reversible block [48]. Cells were grown in a medium containing 4mM thymidine (Sigma-Aldrich, Saint-Louis, MO, USA) for 72 h (first thymidine block), then washed with prewarmed phosphate-buffered saline (PBS) and released for 24 h by culture into a fresh medium. A medium containing 4mM thymidine was added to the cultures for a second 72-h interval (second thymidine block). Finally, the cells were washed and cultured into a fresh medium (T0).

### 4.4. BrdU FACS Analysis

Cell cycle distribution was determined following 2 h of incubation with 30µM 5-bromo-2′-deoxyuridine (BrdU) (Sigma-Aldrich) prior to dissociation with accutase. Cells were washed with PBS and fixed with 70% ethanol at 4 °C for 2h. Fixed cells were permeabilized with 2N HCl and incubated with 0.5 µg/mL Fluorescein isothiocyanate (FITC)—conjugated Mouse Anti-BrdU antibody (BD Biosciences, Franklin Lakes, NJ, USA) or 0.5 µg/mL FITC Mouse IgG1 (BD Biosciences). After 0.25 mg/mL RNase A (Sigma-Aldrich) treatment, cells were stained with 5 mg/mL propidium iodide (BD Biosciences). Cell cycle analysis was carried out on FACS Canto II (BD Biosciences) using FACS Diva software (BD Biosciences). A total of 10,000 events were analyzed for each sample. Cells were collected at 0, 24, 48, 72, and 96 h for fluorescence-activated cell sorting (FACS) (Figure S1).

### 4.5. RI-1 Treatment

RI-1 (Calbiochem, Nottingham, UK) is a chemical covalent inhibitor of the RAD51 protein at cystein 319 preventing subsequent recombinase activity [49]. RI-1 was evaluated at day1, 5, and 9 on GSC-1 (radiosensitive) and GSC-14 (radioresistant) and showed no effect on cell viability at 10 µM (Figure S4). Therefore, GSCs were treated 24 h before IR with 10 μM of RI-1 or Dimethyl sulfoxide (DMSO) vehicle for experiments.

### 4.6. Microarray Analysis

Microarray analysis was performed on an Agilent SurePrint G3 Human GE (8 × 60K) chip (Agilent, Santa Clara, CA, USA). Data were subject to a threshold (values below 1.0 were set to 1.0) and Log2-transformed. Normalization was performed using the per-array and per-gene median scaling method. Genes with very low signals or below noise level were excluded from analysis. All 10 GSCs were analyzed using the 840-gene signature reported by Verhaak et al. [50] and assorted into proneural, mesenchymal, or classical subtypes (Supplementary Table S1). Gene expression of GSCs 1-3-5-10-11 and GSCs 2-9-6-13-14 was compared according to the C5.bp.v6.1 gene set comprising 4436 GO terms and according to a target gene set involved in DNA damage checkpoints, the cell cycle, or DNA repair using Gene Set Enrichment Analysis (GSEA, software.broadinstitute.org/gsea/msigdb/). Lists of two significant enriched signatures are detailed in Table S2.

### 4.7. TaqMan Low Density Array

TaqMan Low density array (Life Technologies) was performed as previously described in [21]. The experiment was conducted in triplicate. A list of 46 genes called “BALBOUS signature” was evaluated (Table S2). Gene expression of GSCs 1-3-5-10-11 and GSCs 2-9-6-13-14 was analyzed according to the 2^−ΔΔ*C*t^ method using *GAPDH* expression as an endogenous control. Descriptive statistics of the results were calculated with GraphPad Prism 6 (La Jolla, San Diego, CA, USA).

### 4.8. Statistical Analysis

Statistical analyses of the results were performed with GraphPad Prism 6 (La Jolla). The results are presented as mean ± standard error of the mean (SEM) and statistical significance was evaluated by nonparametric multiple comparison using the Kruskal–Wallis and Mann–Whitney tests. Δ T-T0 stands for mean variation of cell cycle phases at indicated post-irradiation time points. Statistical group comparison of the TLDA results was evaluated by the nonparametric Mann–Whitney test. *p* values less than 0.05 were considered statistically significant.

## Figures and Tables

**Table 1:** Major differences and characteristics of radiosensitive and radioresistant GSC groups [21].

Data	Variables	Radiosensitive Group	Radioresistant Group
Patient Data	OS	16.8	8.6
	PFS	8	3.4
GSC Data	GSClines	1,3,5,10,11	2,6,9,13,14
	Rad51 expression post-IR 4Gy	Not increased	Increased
	Comet assay post IR 4Gy	DNA breaks	No DNA breaks
	Apoptosis (7 days post-IR 16Gy)	↑40%	↑40%
	Apoptosis (7 days post-IR 16Gy + Rad51 inhibitor)	↑40%	↑75%
	Cell cycle check point post-IR 4Gy	G2 arrest	G2 arrest
	Cell cycle check point post-IR 4Gy + Rad51 inhibitor	Slight decrease G2 arrest	Delayed G2 arrest

OS: Overall Survival; PFS: Progression-Free Survival; GSC lines: Glioma Stem Cell lines; IR: ionizing radiation; arrow ↑: increase.

## Supplementary Materials

Supplementary materials can be found at http://www.mdpi.com/1422-0067/19/10/3018/s1. Figure S1: Representative data from the unsynchronized GSC-1 line showing cell cycle profiles before irradiation (T0) and 24 h post-irradiation (T24). Figure S2: Cell cycle progression of unsynchronized GSC lines up to 72 h after 4Gy IR exposure compared to untreated cells (T0). Table S1: Classification of GSCs using Verhaak signature of 840 genes. Figure S3: Proportion of GSC-14 cells in G2 phase after RI-1 treatment following a 16Gy-IR exposure. Table S2: (Sheet1) Microarray analysis: Ranked list of genes significantly enriched in the radiosensitive group versus theradioresistant group. (Sheet2) Enriched signatures in the radiosensitive group according to the C5.bp.v6.1 gene set. (Sheet3) Enriched signatures in the radiosensitive group according to a target gene set of Go terms involved in DNA damage response. (Sheet4) Gene list of DNA repair TLDA assay: *p* values comparing expression of the radiosensitive group versus theradioresistant group (Mann–Whitney, * *p* < 0.05). Figure S4: Evaluation of RI-1 effect on cell viability at day1 and day2 on GSC-1 (radiosensitive) and GSC-14 (radioresistant).
