# Supplementary figures and images for: Cell Cycle Changes after Glioblastoma Stem Cell Irradiation: The Major Role of RAD51
Source: Int J Mol Sci. 2018 Oct 3;19(10):3018. doi: 10.3390/ijms19103018 (PMC6213228; doi:10.3390/ijms19103018)

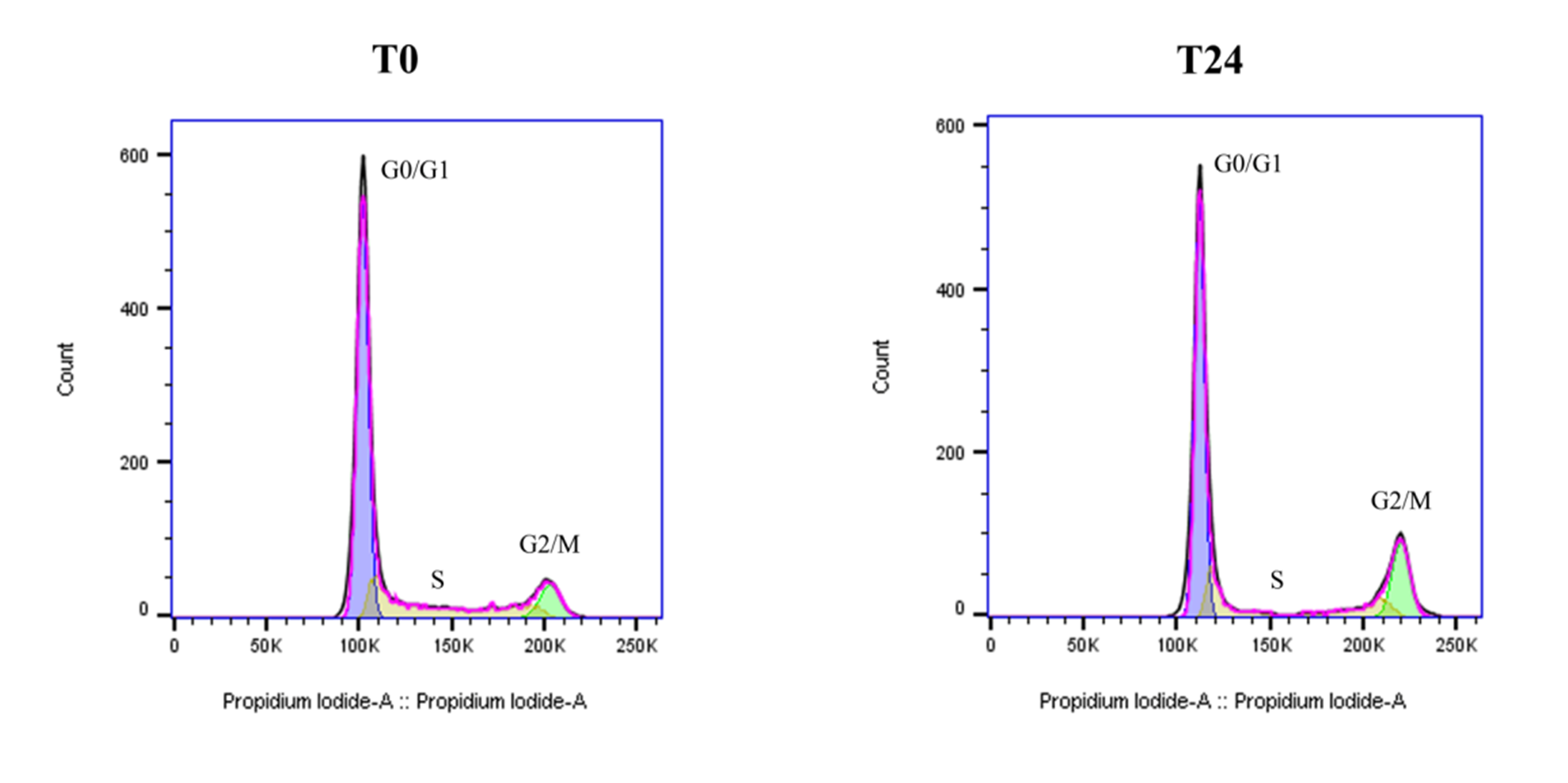

Supplement: Supplementary file 1 [file ijms-19-03018-s001.zip › Suppl. files/Supplementary_file_S1.tif]

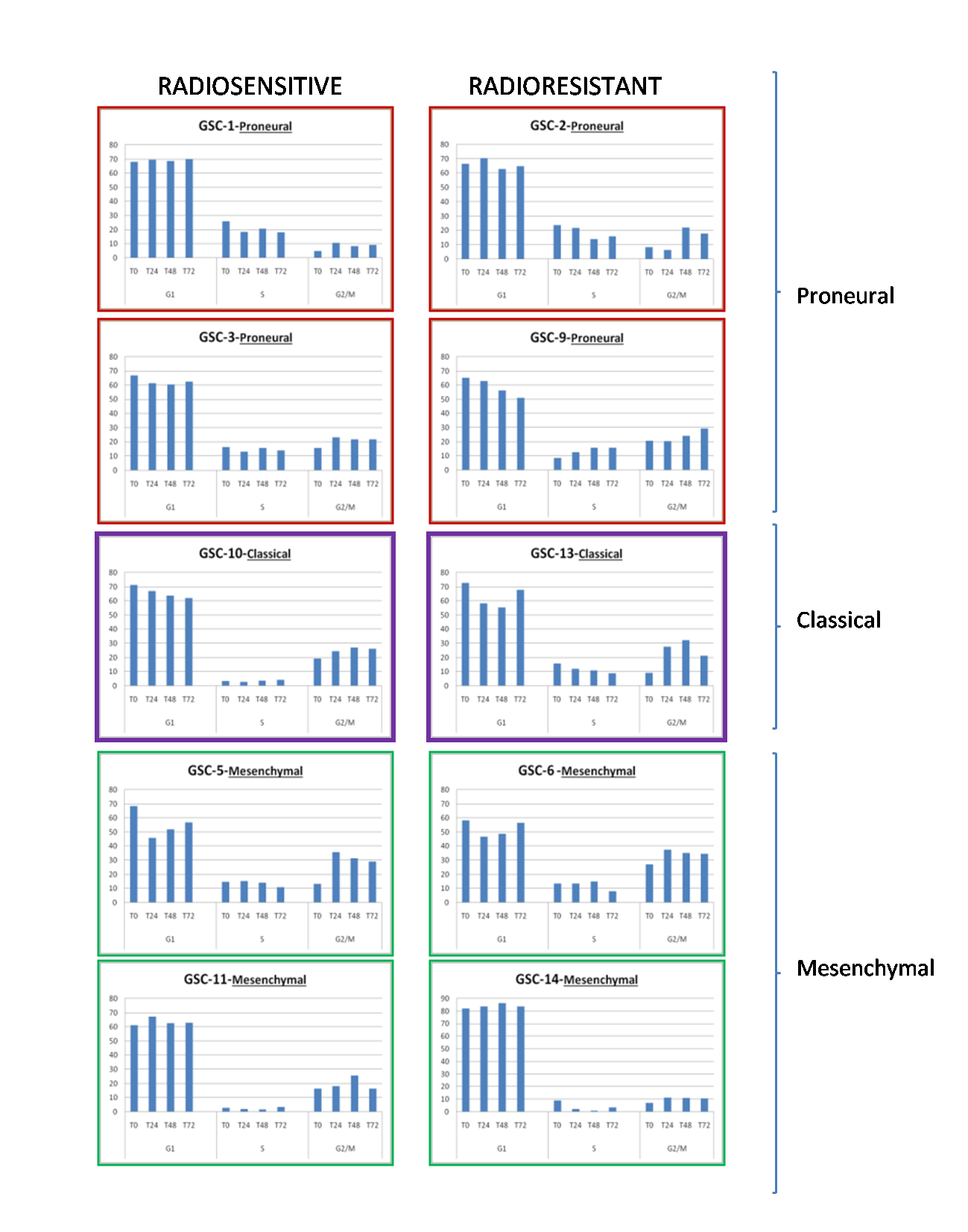

Supplement: Supplementary file 1 [file ijms-19-03018-s001.zip › Suppl. files/Supplementary_file_S2.tif]

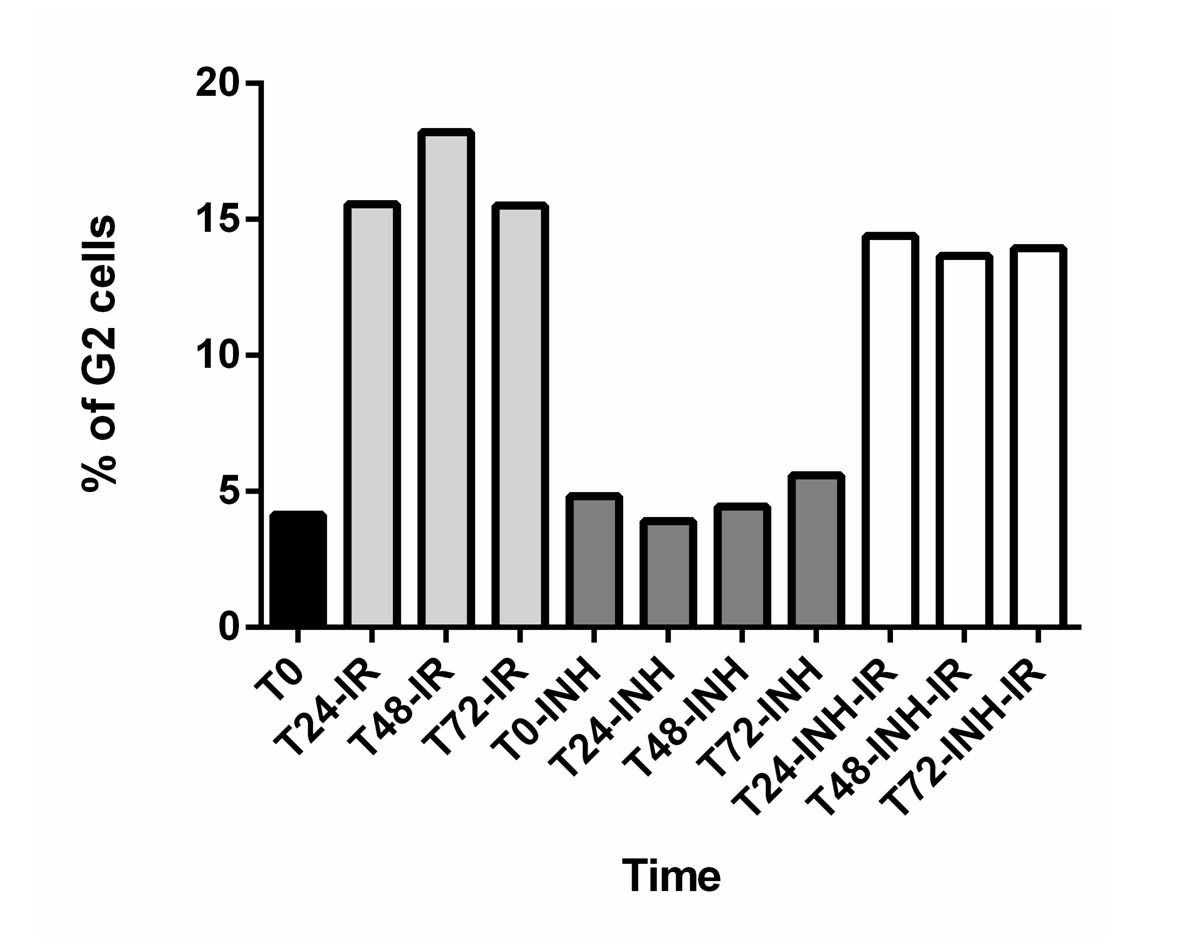

Supplement: Supplementary file 1 [file ijms-19-03018-s001.zip › Suppl. files/Supplementary_file_S3.tif]

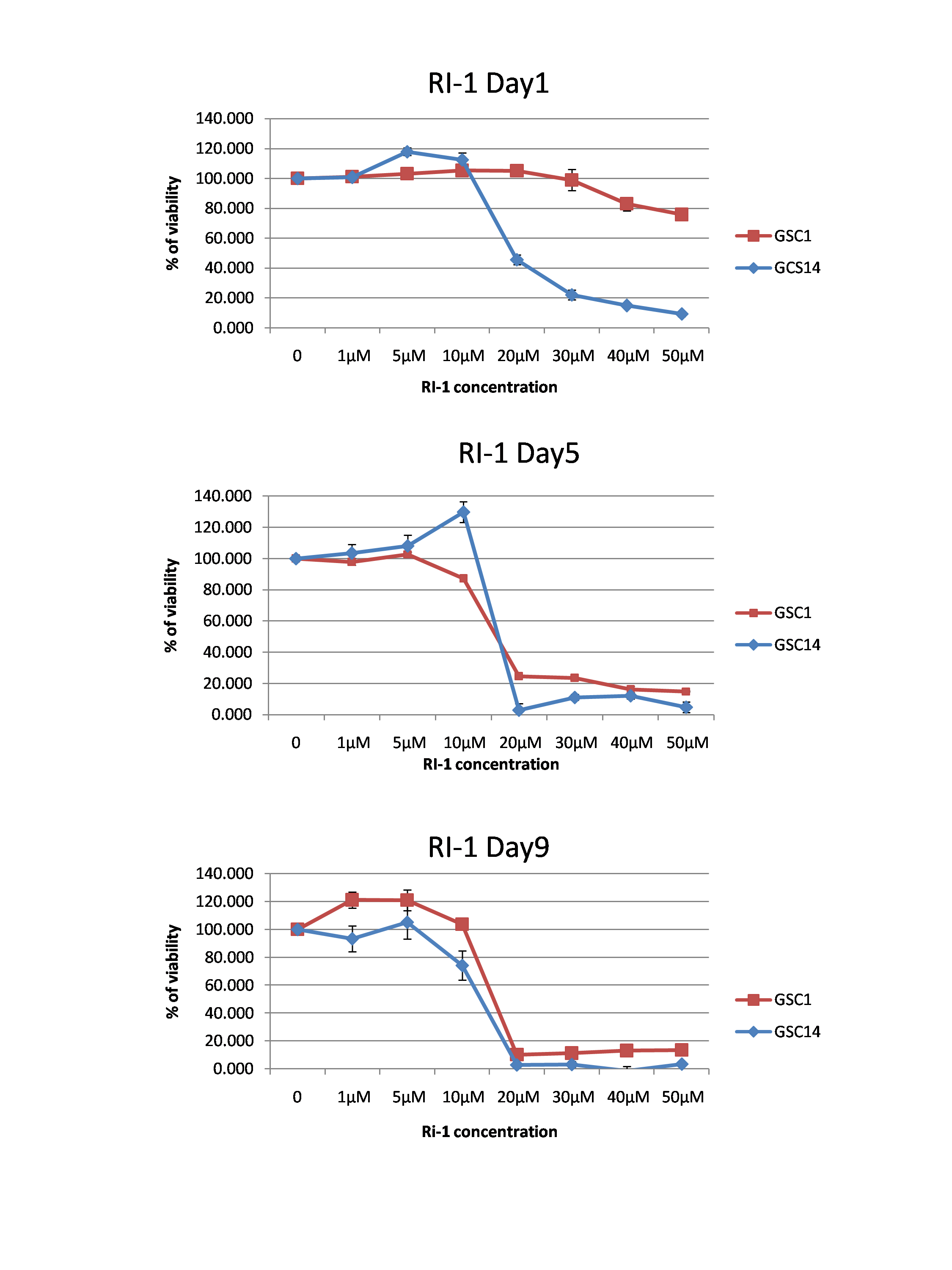

Supplement: Supplementary file 1 [file ijms-19-03018-s001.zip › Suppl. files/Supplementary_file_S4.tif]
